# A Nested Case-Control Study of Intrauterine Exposure to Persistent Organochlorine Pollutants and the Risk of Hypospadias

**DOI:** 10.1371/journal.pone.0044767

**Published:** 2012-09-27

**Authors:** Anna Rignell-Hydbom, Christian H. Lindh, Joakim Dillner, Bo A. G. Jönsson, Lars Rylander

**Affiliations:** 1 Division of Occupational and Environmental Medicine, Lund University, Lund, Sweden; 2 Department of Medical Epidemiology and Biostatistics, Karolinska Institutet, Stockholm, Sweden; Clermont Université, France

## Abstract

**Background:**

Environmental exposures to endocrine disrupting chemicals have been suggested as a risk factor for male genital abnormalities such as hypospadias. The aim of this case-control study was to investigate the association between fetal exposure to persistent organochlorine pollutants (POP) and the risk for hypospadias.

**Methodology/Principal Findings:**

The Southern Sweden Maternity Cohort (SSMC) contains serum samples collected in early pregnancy among women in Southern Sweden. Linkages with the Medical Birth Register, the Malformation Register and the In-patient Register resulted in 390 SSMC mothers who had given birth to a boy with hypospadias in year 1986–2002 (mean 1995). For 237 of these (cases) sufficient amounts of serum for the chemical analyses were available. For each case, a control boy from the SSMC was randomly selected, matched for maternal age, birth year, parity and maternal smoking. PCB-153, *p,p’*-DDE and hexachlorbenzene (HCB) were used as biomarkers for POP exposure. The exposures were categorized into quartiles based on the distributions among the controls. There were no statistically significant trends between the a priori categorisation of the exposure variables and the risk for hypospadias. However, when the upper HCB quartile (>26 ng/ml) was compared to the other quartiles an odds ratio of 1.65 (95% CI 1.02 to 2.69) was obtained. *p,p*′-DDE levels above median (>1.0 ng/ml) compared to levels below 0.1 ng/ml gave an OR of 1.69 (95% CI 0.97 to 2.93).

**Conclusions:**

The present study suggests that fetal exposure to HCB and *p,p’*-DDE may be a risk factor for hypospadias.

## Introduction

In western countries the prevalence of hypospadias varies from two to eight cases per 1,000 live births [1]. This disorder results from an incomplete fusion of the urethral folds between the eighth and 14th weeks of gestation. The male sexual differentiation depends on testosterone, and its metabolite dihydrotestosterone and expression of androgen receptors by target cells [2]. The knowledge about risk factors for hypospadias is still scarce, however environmental exposures to so called endocrine disrupting chemicals (EDCs) have been suggested as a risk factor [3]. These chemicals have the potential to antagonize, enhance or mimic the biological activity of endogenous hormones and disturbance in the balance of the endocrine system may lead to hypospadias [4]–[7]. A large group of EDCs is persistent organochlorine pollutants (POP), such as polychlorinated biphenyls (PCB), dioxins, pesticides such as dichlorodiphenyl trichloroethane (DDT), and hexachlorobenzene (HCB). These chemicals are lipophilic compounds, highly resistant to both abiotic and biotic degradation and they are transported through both watercourses and atmosphere. Although the majority of POPs have been restricted or banned in most countries during the 1970’s and 1980’s, the substances are, due to their persistence, still found in humans [8], [9]. POPs are known to cross the placenta and thereby serve as a prenatal source of exposure for the developing fetus [10], [11].

The major DDT metabolite 1,1-dichloro-2,2-bis (*p*-chlorophenyl)-ethylene (*p,p’*-DDE), which is a strong androgen receptor blocking agent, caused male genital tract malformations in rats [12]. Moreover, abnormal development of Sertoli cells, Leydig cell hyperplasia, decreased testis weight, infertility have been found *in vivo* in animals after exposure to the pesticides *p,p’-*DDE, vinclozolin, fenthion as well as to several phthalates [13]–[17]. Some studies on humans occupationally exposed to EDCs (greenhouse workers and farmers, hairdressers and military personal) have pointed towards an increased risk of genital malformations [1]–[22]. However, in other studies no associations were found [23]–[26].

A recently published epidemiological study from Italy which included 37 serum samples from mothers with boys born with hypospadias indicated a strong association with HCB [27]. The exposures were measured 54 weeks after delivery and not during pregnancy. In Sweden we have accesses to large biobanks were serum samples from pregnancy week 14 have been stored. Due to the unique personal identification number present in the biobank databases and the national health registries in Sweden, we have the possibility to investigate the hypothesized association between POPs and hypospadias in relatively large study.

## Methods

### Study Population and Design

All women were informed that a part of the sample should be stored in a biobank and could be used for research in the future. All participants gave their verbal informed consent at the time blood samples were collected. The study was performed in accordance with the Declaration of Helsinki and approved by the Research Ethics Committee at Lund University.

The Department of Clinical Microbiology, Laboratory Medicine Scania in Malmö is the only virus laboratory serving Southern Sweden. All samples submitted for virus diagnostics have been stored during 1986 and from 1988 onwards, resulting in a biobank that contains about 1,3 million serum samples from about 524.000 subjects. During a reorganization of the biobank unfortunately all samples from 1988 were thrown away. A particularly valuable part of the biobank is samples stored as a result of population-based serological screening for virus infections and rubella immunity during pregnancy (The Southern Sweden Maternity Cohort, SSMC). These samples are scheduled to be taken during week 14 of pregnancy and virtually all pregnant women in the population participate in this screening program. Up to year 2002, the SSMC contained serum samples taken during pregnancies resulting in 101262 single-born boys. A case-control study of the risk of hypospadias in relation to serum levels of POP of the mother during pregnancy was performed of the single-born boys within SSMC.

### Cases

To identify boys with hypospadias within the SSMC, linkages between the Medical Birth Register (MBR), the Malformation Register and the In-patient Register were performed. In the present study boys were defined as hypospadias cases if they were present in two of the three registers, i.e. minor hypospadias were excluded. This resulted in 390 boys with hypospadia. For 237 (61%) of them, sufficient amount of serum samples for the POP analyses were found in the SSMC biobank. Additional information about the pregnancy was retrieved from the MBR (Table 1).

**Table 1:** Descriptive data for the matching variables and other background characteristics among 237 children from the Southern part of Sweden with hypospadias (cases) and 237 matched controls.

	Cases		Controls	
	n	(%)	N	(%)
***Matching variables***				
Birth weight				
1986–1987	18	(8)	18	(8)
1988–1993	73	(31)	74	(31)
1994–1998	65	(27)	62	(26)
1999–2002	81	(34)	83	(35)
Maternal age (years)				
<25	50	(21)	50	(21)
25–29	91	(38)	92	(39)
30–34	61	(26)	60	(25)
>35	35	(15)	35	(15)
Parity				
1	120	(51)	119	(50)
2	77	(32)	76	(32)
>2	40	(17)	42	(18)
Maternal smoking in early pregnancy^a^				
No	180	(81)	183	(82)
Yes	42	(19)	41	(18)
Unknown	15		13	
***Other variables***				
Gestational age (weeks)				
<37	24	(10)	14	(6)
37–38	45	(19)	39	(16)
39–40	114	(48)	122	(52)
>40	54	(23)	62	(26)
Birth weight^b^ (g)				
<2500	21	(9)	9	(4)
>4000	38	(16)	43	(18)
Family situation^c^				
Cohabiting with the father	213	(94)	207	(92)
Else	14	(6)	17	(7)
Working status in early pregnancy^d^				
Full time	104	(51)	106	(52)
Part time	52	(26)	66	(32)
Not working	47	(23)	34	(16)
Caesarean section	32	(14)	30	(13)

a Data missing for 15 cases and 13 controls;

b Data missing for 2 cases and 2 controls;

c Data missing for 10 cases and 13 controls;

d Data missing for 34 cases and 31 controls.

### Controls

For each case a control boy was randomly selected from the SSMC. The aim was to match the controls for maternal age, birth year, parity and maternal smoking habits in early pregnancy, which was successful for almost all controls (Table 1). In addition, boys with cryptorchidism or any other major malformation were excluded.

### Biomarkers of Exposure

In the present study 2,2′,4,4′,5,5′-hexachlorobiphenyl (PCB-153) was selected as a biomarker for PCB exposure as well as dioxin exposure since PCB-153 correlates very well (r = 0.98) with total PCB concentrations in plasma and serum from Swedish individuals and with the TCDD equivalent (TEQ) in plasma from PCB (r = 0.89) [28]–[29]Another POP found in rather high levels in human serum is *p,p’-*DDE, the major DDT metabolite, which was used as a biomarker for DDT and DDE exposure [30] HCB is also a POP found in rather high levels in human serum was used as a biomarker of HCB exposure. There were rather high correlations between the three biomarkers. Using Spearmans correlations rank test PCB-153 was correlated by r_s_ = 0.37 with *p,p*′*-*DDE and r_s_ = 0,51 with HCB. The correlation between *p,p*′-DDE and HCB was r_s_ = 0.52.

### Determination of PCB-153, *p,p*′-DDE and HCB

PCB-153, *p, p*’-DDE and HCB were extracted from 0.05 ml aliquots of serum by solid phase extraction (Strata SDB-L 200 mg; Phenomenex, Torrance, CA, USA) using on-column degradation of the lipids and analysis by gas chromatography mass spectrometry. ^13^C_12_-labeled PCB-153, ^13^C_12_-labeled *p, p*’-DDE and ^13^C_6_-labeled HCB were used as internal standards. The mass fragments monitored were m/z 360, 318 and 284 for PCB-153, *p, p*’-DDE and HCB, respectively. The corresponding mass fragments for the internal standards were m/z 372, 330 and 290, respectively. The relative standard deviations, calculated from 100 samples analyzed in duplicate at different days, was 5% at 2 ng/ml for PCB-153, 8% at 7 ng/ml for *p, p’-*DDE and 6% at 0.3 ng/ml for HCB. The limit of detections were set at 0.05 ng/ml, 0.1 ng/ml and 0.02 ng/ml for PCB-153, *p, p’-*DDE and HCB, respectively. The analyses of PCB-153, *p, p’-*DDE and HCB were part of the Round Robin inter-comparison program (Professor Dr. med. Hans Drexler, Institute and Out-Patient Clinic for Occupational, Social and Environmental Medicine, University of Erlangen-Nuremberg, Germany) with analysis results within the tolerance limits.

### Statistical Analyses

The association between the levels of maternal POP concentrations and the risk for hypospadias in the offspring was evaluated by conditional logistic regression (using the statistical software EGRET), given odds ratios (OR) as the risk measure with 95% confidence intervals (CI). The exposure variables (PCB-153, *p,p’-*DDE and HCB) were categorized into four equally sized groups (≈ quartiles) based on the distributions among the controls. The lowest exposure category was defined as the reference category. We did also estimate p-values for trend by including the categorized exposure variables as continuous variables (coded 0, 1, 2 and 3) in the models. Based on the results where we used the exposure variables categorized into quartiles we performed additional analyses were we merged exposure categories with similar risks. However, we will stress that the results from these latter analyses should be interpreted with caution.

Due to the relatively high correlation between PCB-153, *p,p’*-DDE and HCB concentrations (r_s_ varied between 0.37 and 0.52) we did not include these exposure measures simultaneously in the models. We did, however, create a trichotomized exposure variable based on PCB-153, *p,p*’-DDE and HCB concentrations. The three categories was defined as i) children whose mothers had exposure concentrations in the lowest quartile for all three exposure variables, ii) children whose mothers had exposure concentrations in the highest quartile for all three exposure variables, and iii) all other. Corresponding variable were also created by the use of the median (instead of the lower and upper quartile) as the cut-off level. Moreover, corresponding variables were created by including only two of the exposure variables at a time (i.e. PCB-153 plus *p,p’*-DDE, PCB-153 plus HCB, and *p,p’*-DDE plus HCB, respectively) instead of all three exposure variables.

For three cases and for five controls it was not possible to determine PCB-153 and *p,p’*-DDE concentrations. These eight case-control sets were accordingly only included in the logistic regression models regarding HCB.

## Results

### Exposure Levels

The median maternal serum concentrations of PCB-153, *p,p’*-DDE, and HCB among the cases were 0.45 ng/mL (range 0.03–2.63), 1.24 ng/mL (0.05–23.3), and 0.20 ng/mL (0.01–1.26), respectively. The corresponding figures among the controls were 0.48 ng/mL (0.03–2.24), 1.04 ng/mL (0.05–29.0), and 0.19 ng/mL (0.01–3.25).

### Exposure to a Single POPs and Hypospadias

No statistically significant dose-response associations were observed between maternal serum concentrations of PCB-153, *p,p’*-DDE or HCB (categorized into quartiles) and the risk for hypospadias (p-values for trend: 0.13, 0.09, and 0.12; Table 2). When comparing the highest with the lowest PCB-153 quartile gave an OR of 0.60 (95% CI 0.30 to 1.19; p-value = 0.15).

**Table 2:** Maternal serum concentrations of PCB-153, *p,p’*-DDE and HCB during early pregnancy (around gestational week 12–14), and the risk for hypospadias among their infants.

	Cases	Controls	OR	95% CI	p-value for trend
PCB-153 (ng/mL)					0.13
−0.25 (ref)	68	62	1.00	–	
>0.25–0.48	59	53	0.96	0.57, 1.62	
>0.48–0.76	54	57	0.75	0.42, 1.35	
>0.76	48	57	0.60	0.30, 1.19	
p,p’-DDE (ng/mL)					0.09
−0.10 (ref)	46	59	1.00	–	
>0.10–1.00	52	54	1.33	0.73, 2.44	
>1.00–2.20	67	59	1.69	0.93, 3.08	
>2.20	64	57	1.68	0.92, 3.08	
HCB (ng/mL)					0.12
−0.15 (ref)	63	64	1.00	–	
>0.15–0.19	47	60	0.80	0.46, 1.38	
>0.19–0.26	52	55	1.04	0.59, 1.84	
>0.26	75	58	1.59	0.86, 2.93	

Odds ratios (OR) and 95% confidence intervals (CI) were obtained with conditional logistic regression analysis.

When the upper two quartiles (i.e. above median) of *p,p*′-DDE exposure were merged into one group, and compared with the reference group, an OR of 1.69 (95% CI 0.97 to 2.93: p-value = 0.06, Table 3) was obtained. The risk for hypospadias was statistically significantly increased in the highest exposure quartile of HCB as compared to the three lowest HCB exposure categories (merged into one reference group). The obtained OR was 1.65 (95% CI 1.02 to 2.69; p-value = 0.04).

**Table 3:** Maternal serum concentrations of *p,p′-*DDE and HCB during early pregnancy (around gestational week 12–14), and the risk for hypospadias among their infants.

	Cases	Controls	OR	95% CI
p,p’-DDE (ng/mL)				
−0.10 (ref)	46	59	1.00	–
>0.10–1.00	52	54	1.33	0.73, 2.44
>1.00	131	116	1.69	0.97, 2.93
HCB (ng/mL)				
−0.26 (ref)	162	179	1.00	–
>0.26	75	58	1.65	1.02, 2.69

Odds ratios (OR) and 95% confidence intervals (CI) were obtained with conditional logistic regression analysis.

### Exposure to POP Mixtures and Hypospadias

No statistically significant trends were observed between maternal serum concentrations of POP mixtures and the risk for hypospadias (Table 4). The only observation which was in direction of the hypothesis, although not statistically significant, was the increased risk for hypospadias among cases in the high “*p,p’*-DDE plus HCB” category as compared to the low exposure category (OR 1.86, 95% CI 0.83 to 4.20; p-value = 0.13).

**Table 4:** Maternal serum concentrations of mixtures of PCB-153, *p,p’*-DDE and HCB during early pregnancy (around gestational week 14), and the risk for hypospadias among their infants.

	Cases	Controls	OR	95% CI	p-value for trend
PCB-153+ p,p’-DDE + HCB^a^					1.00
Low (ref)	16	15	1.00	–	
Medium	188	190	0.92	0.42, 2.02	
High	25	24	0.98	0.35, 2.77	
PCB-153+p,p’-DDE^a^					0.74
Low (ref)	26	30	1.00	–	
Medium	173	168	1.24	0.65, 2.35	
High	30	31	1.16	0.49, 2.79	
PCB-153+ HCB^a^					0.69
Low (ref)	37	29	1.00	–	
Medium	153	165	0.69	0.39, 1.25	
High	39	35	0.85	0.38, 1.88	
p,p’-DDE + HCB^a^					0.13
Low (ref)	21	28	1.00	–	
Medium	166	167	1.41	0.74, 2.67	
High	42	34	1.86	0.83, 4.20	

Odds ratios (OR) and 95% confidence intervals (CI) were obtained with conditional logistic regression analysis.

a Low  =  children whose mothers had exposure concentrations in the lowest quartile for all exposure variables, High  =  children whose mothers had exposure concentrations in the highest quartile for all exposure variables, and Medium  =  all other.

## Discussion

No statistically significant dose-response associations were observed between maternal serum concentrations of PCB-153, *p,p’*-DDE or HCB categorized into quartiles and the risk for hypospadias. However, when performing further statistical analyses, the present study indicates that *in utero* exposure to HCB may be a risk factor for hypospadias. Mothers with a serum HCB level above 26 ng/ml were at increased risk to bear a child with hypospadia (OR: 1.65 (95% CI 1.02 to 2.69). Exposure to *p,p’*-DDE tended to be associated with risk. Maternal PCB-153 concentrations showed an decreased risk although not significant.

A recently published study by Giordano and colleagues found a fivefold increased risk of having a boy with hypospadias among Italian women with serum HCB concentrations above 0.16 ng/g (i.e. the median concentration in the study) as compared to women with lower HCB concentrations [27]. To the best of our knowledge no other studies have investigated the association between HCB and hypospadias. The exposure levels in our study were very similar to those in the Italian study. Giordano and colleagues had 37 cases included their study. This means that they only had statistical power to detect large effects, which could be illustrated for *p,p’*-DDE as well as for PCB-153 exposure where risk estimates of 1.81 and 1.91 were far from reaching statistically significance. These estimates were higher as compared to the ones observed in the present study. With 237 cases as in the present study we had the possibility to detect also more moderate effects. Another major difference between the Italian study and the present study were the time when the maternal serum samples were collected. In the Italian study the mean time since birth of the index infant were 53.77 weeks among cases and 34.47 weeks among controls. In contrast to this the samples in our study were collected during a more relevant window, i.e. in the period of masculinization, corresponding to 8–14 week of gestation. The impact of this discrepancy is, however, hard to evaluate.

In recently published studies on PCB concentrations in breast milk and placenta and risk for cryptorchidism, another male genital disorder which share many risk factors with hypospadias, no increased risk of cryptorchidism where seen [31]–[32]. On the contrary, as in the present study there was a tendency to a protective effect. This raises the question whether exposure to POPs have a protective effect. An explanation could be that in Sweden, fish intake is associated with higher levels of POP biomarkers in blood. These biomarkers might, however, not only be a proxy for the toxic compounds, but also for the essential long-chain fatty acids eicosapentaenoic acid (EPA) and docosahexaenoic acid (DHA) present in fatty fish which might act as protective [33].

However, Damgaard et al found a positive association between chlorinated pesticides and cryptorchidism [34].

The registers in Sweden are almost complete (98%) and by the use of different registers we will most probably miss very few hypospadias cases. A possible weakness is that the material may be skewed to severe cases that required operative treatment and the mild cases have not been included. It has been postulated that environmental factors would be particularly important in the pathogenesis of these cases, whereas the severe cases with proximal hypospadias may be more often associated with genetic defects. However, these cases of hypospadias are very few.

In addition, information about potential confounders is available from the MBR. On the other hand did we lack or did not have sufficient amount of maternal serum for almost 40% of the cases. It is, however, not reasonable to believe that this selection is due to POP levels. The fact that the distributions for the few socio-economic variables we had information about, i.e. family situation and working status in early pregnancy, were very similar among the cases and the controls do not support that selection bias is a major problem in the present student. Another possible limitation in the present study was the small amount of serum available from the biobank. The chemical analyses were therefore restricted to PCB-153, *p,p’*-DDE and HCB. However, despite the small volume of serum available the precisions were high and the detection limits sufficiently low to obtain results above the detection limits in most samples. Furthermore, the agreements of the results obtained by the current method with results from an inter-laboratory comparison program indicate a high accuracy. On the other hand, because of the small volumes available we did not have the possibility to lipid adjust our samples. However, we do not see this as a major problem due to the very strong correlations (r>0.90) found between fresh and lipid adjusted PCB-153 concentrations found in a study among women from Sweden.

The foetal period is a vulnerable period for toxic substances. POPs can pass the placental barrier and most POPs have very long half-lives, five to ten years. It is then reasonable to believe that POP levels measured in serum women reflect the *in utero* exposure in the fetus.

### Conclusions

The hormone hypothesis of male reproductive disorders suggests that both endogenous and exogenous hormones might be a risk factor for hypospadias. Endocrine disrupting chemicals, such as PCBs, *p,p’*-DDE and HCB, may alter hormone levels and thereby affect the fetus.

The present study indicates that *in utero* exposure to HCB and perhaps exposure to *p,p’*-DDE is a risk factor for hypospadias. The fact that HCB and *p,p’*-DDE may affect the androgen-signalling pathway should reinforce the concept that environmental xenobiotics, though present at low doses, may affect human health negatively.
